# Fitness Effects of Spontaneous Mutations in Picoeukaryotic Marine Green Algae

**DOI:** 10.1534/g3.116.029769

**Published:** 2016-05-10

**Authors:** Marc Krasovec, Adam Eyre-Walker, Nigel Grimsley, Christophe Salmeron, David Pecqueur, Gwenael Piganeau, Sophie Sanchez-Ferandin

**Affiliations:** * Sorbonne Universités, UPMC Univ Paris 06, CNRS, Biologie Intégrative des Organismes Marins (BIOM), Observatoire Océanologique, F-66650 Banyuls/Mer, France; † Sorbonne Universités, UPMC Univ Paris 06, CNRS, Observatoire Océanologique de Banyuls (OOB) , F-66650 Banyuls/Mer, France and; § School of Life Sciences, University of Sussex, Brighton BN1 9QG, United Kingdom

**Keywords:** spontaneous mutation, mutation accumulation, fitness effects, marine pico-phytoplankton, single cell cultures

## Abstract

Estimates of the fitness effects of spontaneous mutations are important for understanding the adaptive potential of species. Here, we present the results of mutation accumulation experiments over 265–512 sequential generations in four species of marine unicellular green algae, *Ostreococcus tauri* RCC4221, *Ostreococcus mediterraneus* RCC2590, *Micromonas pusilla* RCC299, and *Bathycoccus prasinos* RCC1105. Cell division rates, taken as a proxy for fitness, systematically decline over the course of the experiment in *O. tauri*, but not in the three other species where the MA experiments were carried out over a smaller number of generations. However, evidence of mutation accumulation in 24 MA lines arises when they are exposed to stressful conditions, such as changes in osmolarity or exposure to herbicides. The selection coefficients, estimated from the number of cell divisions/day, varies significantly between the different environmental conditions tested in MA lines, providing evidence for advantageous and deleterious effects of spontaneous mutations. This suggests a common environmental dependence of the fitness effects of mutations and allows the minimum mutation/genome/generation rates to be inferred at 0.0037 in these species.

Mutations are the main drivers of genetic diversity that enable species to adapt by natural selection. Estimating the spontaneous mutation rate and the fitness effects of mutations is, thus, essential for a better understanding of the evolution and the adaptive potential of species (Wright 1932; Kondrashov 1988). A proportion of new mutations are deleterious (Charlesworth and Charlesworth 1998; Keightley and Lynch 2003; Lynch *et al.* 1999), and some of the strongest evidence for this comes from mutation accumulation (MA) experiments, pioneered by Mukai in *Drosophila melanogaster* (Mukai 1964). The accumulation of mutations can be measured experimentally by monitoring the growth, or other fitness traits, of independent lines starting from one genotype for a given number of generations (see Halligan and Keightley 2009 for a review). Serial bottlenecks make natural selection ineffective in the face of genetic drift and permit deleterious mutations to segregate and become fixed in MA lines. Since Mukai’s first experiments in *Drosophila*, many MA experiments have been performed in different organisms: *Arabidospis thaliana* (Shaw *et al.* 2000), *Caenorhabditis elegans* (Ajie *et al.* 2005; Katju *et al.* 2014; Vassilieva *et al.* 2000; Vassilieva and Lynch 1999), *Daphnia pulex* (Deng *et al.* 2002; Deng and Lynch 1997; Schaack *et al.* 2013), *Dictyostelium discoideum* (Hall *et al.* 2013), *D. melanogaster* (Fernández and López-Fanjul 1996; Fry 2004, 2001; Fry *et al.* 1999; Keightley 1994; Schrider *et al.* 2013), *Saccharomyces cerevisiae* (Wloch *et al.* 2001; Zeyl and DeVisser 2001), and *Tetrahymena thermophila* (Long *et al.* 2013). Generally, these experiments show a decrease of fitness in the MA lines as the experiment progresses, consistent with a substantial proportion of spontaneous mutations being deleterious.

MA experiments also enable the relationship between the fitness effects of mutations and the environment to be explored. Knowledge about genotype–environment (GxE) interactions is essential to understand the adaptation process, because fitness effects of mutations may change with time and spatial scales. In *D. melanogaster* (Fry *et al.* 1996; Kondrashov and Houle 1994), *C. elegans* (Baer *et al.* 2006) or *S. cerevisiae* (Korona 1999), the fitness effects of spontaneous mutations change with environmental conditions. However, this interaction is not systematic; in the case of *A. thaliana*, one experiment showed a positive GxE interaction in fitness effects of mutations (Rutter *et al.* 2012), whereas other studies did not (Chang and Shaw 2003; Kavanaugh and Shaw 2005). The nature of the change in mutational effect with environmental conditions allows us to infer three biological implications (Martin and Lenormand 2006): (i) a change in the genomic mutation rate *U* can be interpreted as changes in the expression of mutated genes, (ii) an increase of the fitness variance suggests a variation in the fitness effects of mutation between environments (iii), a change in the average fitness measured might be explained by increased selection strength in harsh conditions.

In harsh environments, the effects of deleterious mutations are expected to increase, because of the biological and ecological pressure induced by stress. However, this view is disputed by experimental evidence in *Escherichia coli* (Kishony and Leibler 2003) and *C. elegans* (Andrew *et al.* 2015). In general, the interaction between stress and fitness effects of mutations may be categorized as follows (Elena and de Visser 2003): first, unconditionally deleterious, with the magnitude of the stress increasing the deleterious effect; second, conditionally neutral, *i.e.*, neutral in some conditions and deleterious in others; third, conditionally beneficial, *i.e.*, advantageous in some conditions but deleterious in others.

While most MA experiments have been performed in model organisms, no results are available in marine phytoplanktonic eukaryotes. Here, we report MA experiments in four haploid marine green algae (Chlorophyta): *Ostreococcus tauri* RCC4221 (Blanc-Mathieu *et al.* 2014), *Ostreococcus mediterraneus* RCC2590 (Subirana *et al.* 2013), *Micromonas pusilla* RCC299 (Worden *et al.* 2009), and *Bathycoccus prasinos* RCC1105 (Moreau *et al.* 2012). All species belong to the Mamiellales order (class Mamiellophyceae, Marin and Melkonian 2010), and are widespread members of the marine phytoplankton (De Vargas *et al.* 2015) that sustain the marine ecosystem in coastal areas (Worden *et al.* 2004). These green algae contain the smallest known free-living eukaryotes (Courties *et al.* 1994), defined as the pico-phytoplankton (see Massana 2011 for a review). They have a simple cell organization, with only one chloroplast and one mitochondrion, and a small genome of 13–21 Mb. 

## Materials and Methods

### Biological models

We performed MA experiments on four haploid marine green algae (Chlorophyta): *O. tauri* RCC4221, *O. mediterraneus* RCC2590, *M. pusilla* RCC299, and *B. prasinos* RCC1105. All cultures are available from the Roscoff Culture Collection (http://roscoff-culture-collection.org/). The identity of each strain was confirmed by 18S rDNA sequencing and PFGE migration (Schwartz and Cantor 1984) at the start of the experiment. All species were kept in L1 liquid medium (salinity of 35 g/L) with a light:dark (LD) cycle of 8:16 (8 hr light 16 hr dark) in 24-well plates, at 20°, except for *B. prasinos* RCC1105, for which the cycle was 12:12 LD.

### MA experiments

**Figure 1:**
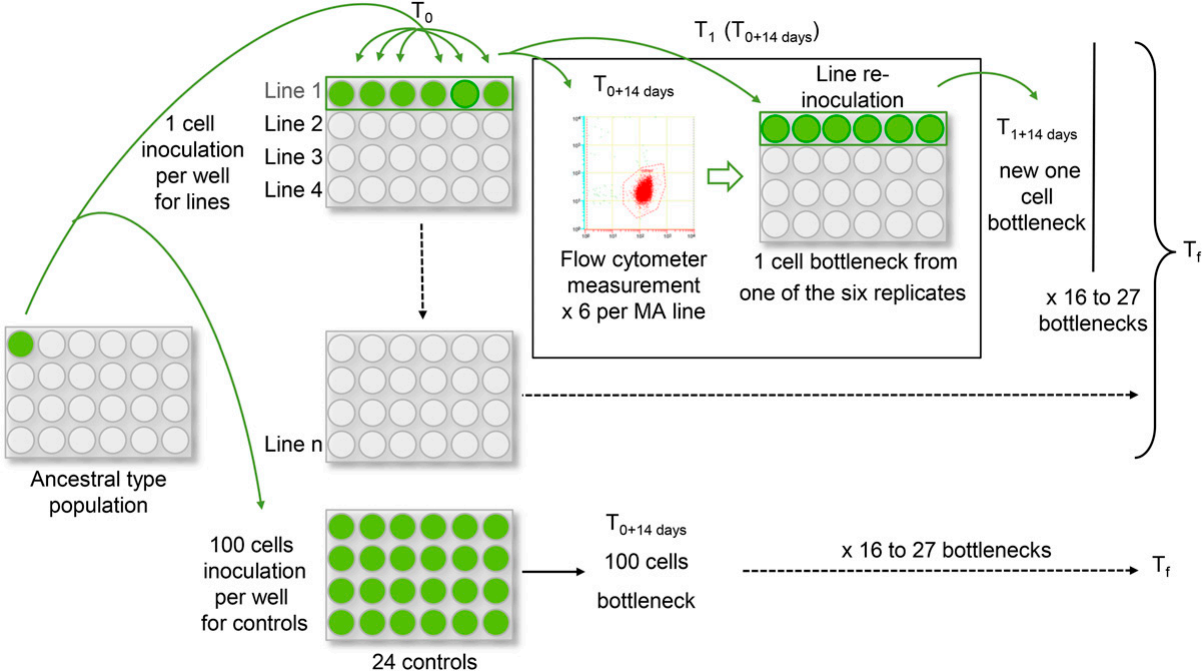
Mutation accumulation (MA) experiments in pico-algae. Flow cytometer measurements were performed every 14 d to make one cell bottlenecks for each line. The ancestral culture of each species came from one single cell, inoculated in a well to grow enough cells to start the experiment. The ancestral culture was maintained with higher effective population size in the control lines (inoculation of 100 cells) and MA lines by reinoculating one single cell, in six replicates per line, in 24-well microtiter plates.

Each experiment was started with one single cell, which divided to produce the ancestral population, from which single cells were sampled to generate independent lines by one cell inoculation (Figure 1). For each species, we inoculated 40 MA lines, kept in 24-well microtiter plates. As a control, the ancestral population was cultured in the same conditions, but with an inoculation of 100 cells, to maintain a larger effective population size. We kept one microplate of controls, *i.e.*, 24 control replicates.

Classically, in MA experiments of unicellular organisms, a colony of cells is transferred to a fresh agar plate at each bottleneck to allow the separation of the cells and the random sampling of a new cell. However, this is not possible in these species as they do not grow on the surface of gelled media, and only grow slowly within gelled medium, in contrast to *S. cerevisiae*, *D. discoideum* or *Chlamydomonas reinhardtii* (Hall *et al.* 2013; Morgan *et al.* 2014; Wloch *et al.* 2001). Nevertheless, they are easily cultured in liquid medium in the laboratory. Therefore, we developed an experimental protocol combining flow cytometry, which has the advantage of counting individual cells while verifying cell size and fluorescence, and transfer of single cells in liquid media. Bottlenecks of MA lines to one cell were performed every 14 d. However, since the number of sampled cells follows a Poisson distribution, the probability of line loss by sampling one single cell is 0.37. Indeed, in contrast with agar plate protocols, a colony cannot be observed in liquid medium, and the cell densities were never large enough to be seen as green. Thus, we measured the number of cells in our wells and calculated the volume needed to extract 10 cells, from which we sampled six for the next new six wells with fresh media. Thus, we maintained six replicates per line at each bottleneck.

If we assume that cells are uniformly distributed through the medium, the number of sampled cells, *N*, is Poisson distributed:

$$P\left(N;\bar{N}\right)=\frac{e^{-\bar{N}}{\bar{N}}^{N}}{N!}$$ (1)

We inoculated those cells into a volume *V* from which we drew aliquots such that we ultimately discarded a proportion *q* of the sample. For a particular sample, the probability that all *N* cells are discarded is simply *q^N^*. Thus, the overall probability that we discard all cells and hence lose a line is:

$$G=\sum_{N=0}^{\infty }P\left(N;\bar{N}\right)q^{N}$$ (2)

If we wanted to include pipetting error, we could model this by assuming that the volume sampled differs from that intended by a factor α which is γ distributed with a mean of 1 and a shape parameter of β. Now equation 1 becomes:

$$P\left(N;\bar{N},\beta \right)=\int_{\alpha =0}^{\infty }\frac{e^{-\alpha \bar{Ν}}{\left(\alpha \bar{N}\right)}^{N}}{N!}D\left(\alpha ;\beta \right)d\alpha$$ (3)

This is actually a negative binomial:

$$P\left(N;\bar{N},\beta \right)=\frac{1}{N!\Gamma \left(\beta \right)}{\bar{N}}^{N}{\left(\frac{1}{\beta }\right)}^{\beta }{\left(\bar{N}+\beta \right)}^{-N-\beta }\Gamma \left(N+\beta \right)$$ (4)

So the probability of observing *k* or more line losses over *t* transfers is given by multiplying *G* from equation (2) by *k* and *t*.

One fifth of the microtiter plate’s volume was used for Cell counting, using a FACSCanto II flow cytometer (Becton Dickinson, Franklin Lakes, NJ) equipped with an air-cooled laser providing 15 mW at 488 nm with the standard filter set-up. Becton Dickinson Trucount^TM^ beads were used to calculate the abundance of the cells as described by Pecqueur *et al.* (2011). A total of 20 µl of mixed fluorescent beads 1 µm in diameter (Molecular Probes Inc., Eugene, OR) were added as an internal standard to 300 µl of the diluted sample (20th dilution). The flow rate of the cytometer was set to high (acquisition time: 1 min). Eukaryotic pico-phytoplankton cells were detected and analyzed using natural chlorophyll fluorescence (chlorophyll a FL3 670 nm LP). The flow cytometry data were analyzed using BD FACSDiva (Becton Dickinson).

In total, the experiments involved 27 bottlenecks over 378 d for *O. tauri*, 21 bottlenecks over 294 d for *O. mediterraneus*, 21 bottlenecks over 302 d for *M. pusilla*, and 16 bottlenecks over 224 d for *B. prasinos* (Table 1).

**Table 1:** Summary of mutation accumulation experiments for four species

Species	Number of Lines	Average Number of Generations Per Line	N_e_	T_0_–T_f_ (d)
*O. tauri* RCC4221	21	512	8	378
*O. mediterraneus* RCC2590	24	272	6	294
*M. pusilla* RCC299	7	272	6	302
*B. prasinos* RCC1105	8	265	8	224

The number of lines is the number of surviving independent lines since the start of the experiment (*T_0_*) to the end (*T_f_*). *N_e_* is the average of effective population size between each bottleneck. The last column is the total duration of the experiment. The probability of line loss was estimated using equation (2) in the *Materials and Methods* section, *N* = 10, and *q* = 0.4. Expected number of line losses (*L_exp_*) is estimated for each species as a function of the coefficient of variation in sampling cells (Table 2).

### Estimation of fitness

We estimated the fitness of lines from the number of divisions/day, *G*, calculated over a period of 14 d using the equation:

$$G=e^{\left[\ln\left(N_{t}/1\right)/t\right]}$$ (5)

*N_t_* is the final number of cells just before the bottleneck and *t* = 14 the number of days between two bottlenecks (*t* = 14). *G* is the number of generations/day. To compare *G* between different MA lines over time, the relative fitness, *G_r_* (*G_r_* = *G_MA_*/*G_control_*), was computed. The effective population size of MA lines and control line populations at each bottleneck was estimated as the harmonic mean of the population size between *t* = 1 to *t* = 14 days. Following Chevin (2011), the fitness effects of mutations in the MA lines at the end of the experiment were measured by estimating the selection coefficient scaled by the generation time, *S_T_*.

$$S_{T}=\frac{\ln\left(G_{MA}\right)-\ln\left(G_{control}\right)}{\ln\left(G_{control}\right)}\ln2$$ (6)

### Fitness assays in stressful conditions

Upon completion of the MA experiments in *O. mediterraneus*, *M. pusilla*, and *B. prasinos*, we used MA lines that had survived from the first to the last generations in each species for further investigations in stressful conditions: nine MA lines of *O. mediterraneus*, seven MA lines of *M. pusilla*, and eight MA lines of *B. prasinos*. For *O. mediterraneus*, 24 MA lines reached the end of the experiment, of which nine were chosen randomly for practicality.

Before starting fitness assays, we transferred MA lines in L1 medium flasks and let them grow for 2 wk to have enough cells to inoculate cultures. Fitness assays were performed in 48-well microtiter plates, with a starting population of ∼50,000 cells/well. For herbicide tolerance tests, we used Diuron at 10 μg/L and Irgarol 1051 at 1 μg/L (Sanchez-Ferandin *et al.* 2013). We tested salinities of 5, 20, 35, 50, and 65 g/L using L1 medium supplements (Guillard and Hargraves 1993). The number of biological replicates was three for each MA line and four for each control. Cell concentrations were obtained by flow cytometry 7 d after plate inoculation and *S_T_* was estimated as specified above. This corresponds to a total of 52 wells measures for *M. pusilla*, 58 for *B. prasinos*, and 64 for *O. mediterraneus*. In *O. tauri*, the MA experiment was completed 6 months before the start of the fitness assays under stressful conditions, so fitness assays could not be performed for this species.

### Statistical analysis

First, to investigate the relationship between fitness, *G*, and the number of sequential generations, we used data from those lines that survived throughout the experiment: 21 lines for *O. tauri*, 24 for *O. mediterraneus*, eight for *B. prasinos*, and seven for *M. pusilla*. We performed an ANOVA on the control data to test whether *G* changed significantly between bottleneck times. The change in fitness of MA lines as a function of time was thereafter analyzed by dividing the growth rate in the MA lines by the growth rate in the control, *G_r_*, to remove the variation in the experimental set-up through time. For each line, the relationship between the relative fitness (*G_r_*) and the number of generations was tested using Pearson’s correlation.

Second, for fitness assays in stressful conditions, *S_T_* was calculated in all conditions using *G*_*MA*_ and *G*_*control*_ at each condition as explained above. We used a pairwise Student’s test to detect changes between MA lines and control. The p-value was corrected for multiple testing using the Bonferroni-Holm method (Holm 1979), as implemented in R. Because MA lines could have fixed more than one mutation during MA experiments, the selection coefficient is estimated for a potential set of mutations, including their possible epistatic effects on fitness.

To check that the environmental assays were indeed stressful for our cultures, *G*_*control*_ of the 24 controls at the end of the MA experiment was compared to *G*_*control*_ of the four controls in each of the environmental conditions. A significant decrease of *G* in an environmental condition confirmed its stressful effect.

Finally, the salinity of 35 g/L is the standard salinity of culture. We performed a Fisher-Snedecor test to detect changes in variance between the standard salinity and the other salinities.

Statistical analyses were performed with R (version 3.1.1) (R Core Team 2014).

### Data availability

Supplemental Material, Table S1, Table S2, Table S3, and Table S4 contain fitness data of each MA line during the experiments. Table S5, Table S6, and Table S7 contain fitness data for fitness assays in herbicides and salinity gradient conditions. Control data during MA experiments are provided in Table S8, Table S9, Table S10, and Table S11.

## Results

### MA experiments

The average effective population sizes across the experiment were six cells for *O. mediterraneus* and *M. pusilla* and eight cells in *B. prasinos* and *O. tauri* (Table 1). The effective population size in the control, which was started with an initial cell number of 100, was estimated to be 600 for *M. pusilla*, 650 for *O. mediterraneus*, and 700 for the other two species. Between each bottleneck, depending on species and lines, the lines divided 10–20 times, corresponding to 512 independent sequential generations/line for *O. tauri*, 272 for *O. mediterraneus*, 265 for *B. prasinos*, and 272 for *M. pusilla*, on average (Table 1).

### Fitness effects of mutations during the MA experiment

We measured the fitness of our MA lines as the number of cell divisions that occurred between two bottlenecks. There was no increase or decrease in the growth rate of the control lines with generation time, but there was a significant variation between bottleneck times (ANOVA, p-value < 0.001) for all species. The fitness values of MA lines were thus divided by the mean fitness estimation of the control, *G_control_*, to yield relative fitness values, *G_r_*; this was done to eliminate any changes in fitness due to uncontrolled variation in the experimental set-up.

The average *G_r_* of *O. tauri* MA lines per bottleneck event decreases significantly with time (Pearson correlation test, ρ = −0.49, p-value = 0.047). Also, four independent MA lines of the 21 had an individually significant decrease of *G_r_* (Pearson correlation test; ρ = −0.54, p-value = 0.026; ρ = −0.51, p-value = 0.035; ρ = −0.56, p-value = 0.018; and ρ = −0.55, p-value = 0.022) (Table S4).

In *O. mediterraneus*, *G_r_* significantly increased in one line (Pearson correlation test, ρ = 0.52, p-value < 0.05). This line is the only one with a significant increase in fitness. No significant increase or decrease of within-species fitness variation of *G_r_* was detected for *M. pusilla* (Table S1), *B. prasinos* (Table S2), and *O. mediterraneus* (Table S3).We also investigated whether the number of lines lost varied over the course of the experiments: the data are consistent with a constant line loss over the course of the experiments in all four species. However, the observed number of lines lost was higher than expected by chance for a coefficient of variation in sampling error equal or smaller to 5% (Table 2) in all species.

**Table 2:** Statistical probabilities of line loss

CV	p	*O. tauri*		*O. mediterraneus*		*B. prasinos*		*M. pusilla*	
		*L_exp_*	*P(L ≥ L_obs_)*	*L_exp_*	*P(L ≥ L_obs_)*	*L_exp_*	*P(L ≥ L_obs_)*	*L_exp_*	*P(L ≥ L_obs_)*
0	0.0025	2.7	0	2.1	0	2.4	0	1.7	0
0.05	0.0026	2.8	0	2.2	0	2.5	0	1.8	0
0.4	0.0150	16.2	0.09	12.6	0	14.4	0	10.2	0
0.5	0.0260	28.1	0.89	21.8	0.5	25.0	0.0000	17.7	0.0033

Statistical probabilities of line loss, with *p* the probability of line loss at each bottleneck, *L_exp_* the expected number of line losses for each experiment, and *L_obs_* the number of observed line losses. Probability of observing *L_obs_* or more line losses, as a function of the number of lines, the number of bottlenecks, *t* (16, 21, and 27 bottlenecks depending on species), and the coefficient of variation of the sampling error (γ distribution with average 1 and Coefficient of Variation *CV*). As an example, for *O. tauri*, the probability of obtaining the observed line loss, *L_obs_*, over the number of bottlenecks performed, with a *CV* of 0.04, is 0.09 [*P(L ≥ L_obs_*)], the expected line loss, *L_exp_*, being 2.8.

### Fitness effects in stressful conditions

#### Herbicide stress:

Both herbicides significantly decreased fitness in the control lines in all tested species when compared to those cultured without herbicide (Wilcoxon test, p-value < 0.001); the herbicides reduced growth rate by 52% and 74% for *B. prasinos*, 40% and 42% for *M. pusilla*, and 52% and 48% for *O. mediterraneus*, in Irgarol 1051 and Diuron media, respectively. In some cases, the variance significantly increased in MA lines (Fisher-Snedecor test, p-value < 0.05 in Irgarol 1051 for *O. mediterraneus* and *M. pusilla*; p-value < 0.001 for *B. prasinos* with the two herbicides). A change of variance is as expected in stressful conditions, because of the revelation of mutation effects.

**Figure 2:**
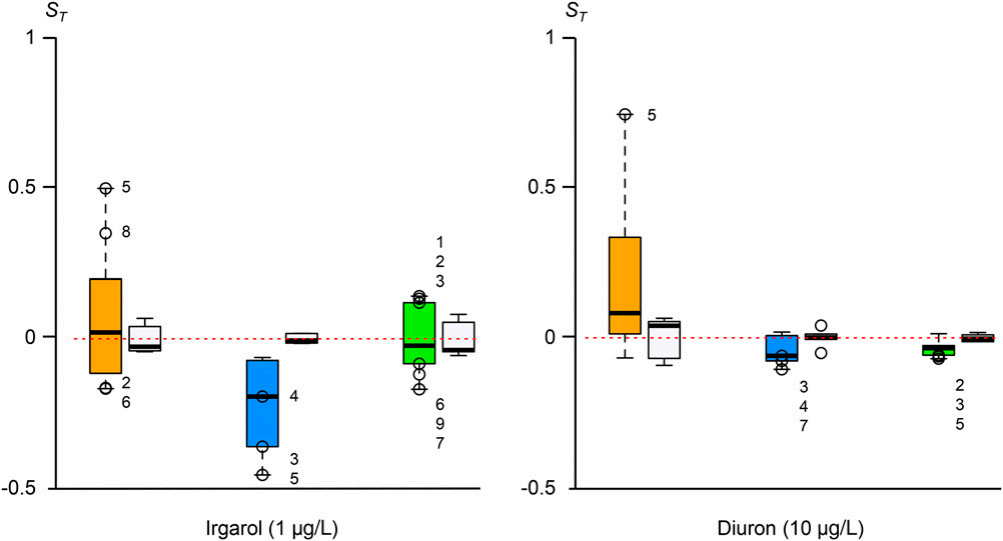
Selection coefficients, *S_T_*, in media containing Irgarol 1051 or Diuron herbicides. Empty circles with a number: MA lines with significant *S_T_* differences (Student’s test, p-value < 0.01). Left to right in the two graphs: *B. prasinos* in orange (eight MA lines), *M. pusilla* in blue (seven MA lines), and *O. mediterraneus* in green (nine MA lines). The *S_T_* of controls are presented as white plots on the left of the MA lines. MA, mutation accumulation.

For each species, the selection coefficients, *S_T_*, are shown in Figure 2. In contrast with the MA experimental conditions, some MA lines showed significantly lower or higher fitnesses with a significant negative or positive selection coefficient. In addition, *S_T_* changed between the two conditions for some identical MA lines.

In all, one MA line had a significantly positive selection coefficient, while two MA lines had a significantly negative selection coefficient in the two conditions.

In summary, out of 24 tested lines, 12 lines (50%) had a significantly negative *S_T_* in at least one herbicide, whereas five lines (21%) had a significantly positive *S_T_*.

#### Osmolarity stress:

MA and control lines were exposed to lower (salinities of 5 and 20 g/L) and higher (salinities of 50 and 65 g/L) levels of salinity than the seawater of their natural environment (35 g/L). Below, we define an environment as stressful if the controls grow more slowly in this environment than in standard conditions, the magnitude of stress being estimated by the growth rate reduction. Both high and low salinities are stressful for *B. prasinos*. In contrast, the control lines of both *M. pusilla* and *O. mediterraneus* grew faster in the slightly lower salinity treatment (20 g/L), and *O. mediterraneus* also grew faster in the lowest salinity treatment (5 g/L) than in the standard conditions (35 g/L), suggesting that lower salinity is not necessarily stressful. A change in the selection coefficient of MA lines is thus not necessarily a consequence of a stress, but just due to benign changes of an environmental parameter.

Stress may be expected to increase the fitness variance. To test this, we compared the variance of *S_T_* in each condition with the standard conditions (35 g/L). The variance of the fitness of MA lines was significantly higher for *O. mediterraneus* in the higher salinity, the most stressful condition (p-value < 0.01). This was also the case for *B. prasinos* in the two higher and lower salinities (p-value < 0.001) and at 20 g/L (p-value < 0.05). In contrast, we did not detect any significant change of the variance in the fitness of *M. pusilla* MA lines between tested conditions.

**Figure 3:**
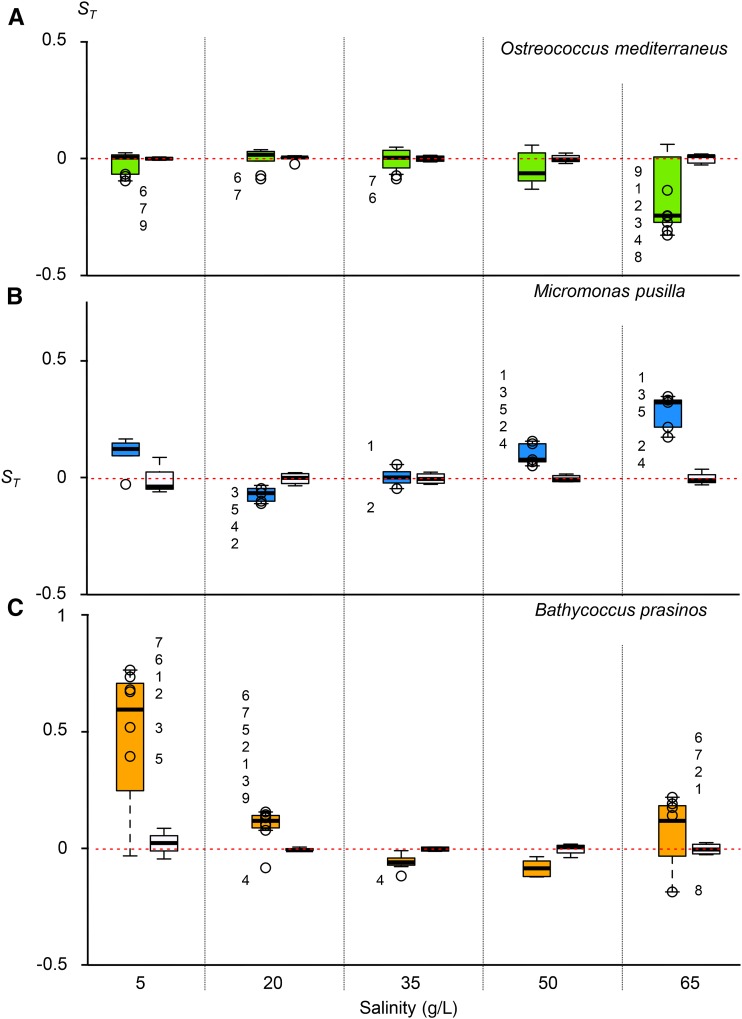
Selection coefficients in five salinity conditions. Empty circles with number are MA lines with significant differences to controls (Student’s test, p-value < 0.01). (A) *O. mediterraneus* in green, nine MA lines. (B) *M. pusilla* in blue, seven MA lines. (C) *B. prasinos* in orange, eight MA lines. The *S_T_* of controls are presented as white plots on the left of the MA lines. MA, mutation accumulation.

The three species showed contrasting patterns in terms of the direction of selection coefficient variation, estimated from the number of cell divisions/day (Figure 3). In *O. mediterraneus*, *S_T_* was systematically negative for the MA lines. In particular, the decrease of *S_T_* was the most significant in the highest salinity, which was the most stressful. *B. prasinos* and *M. pusilla* were much more variable. In *B. prasinos*, almost all MA lines had a significantly higher fitness than the control under stressful conditions, whereas in *M. pusilla* approximately half of the lines with significantly different fitness to the control had higher fitness, and half had lower fitness. Strikingly, the MA lines in *B. prasinos* with higher fitness under low salinity also had higher fitness in higher salinity.

In conclusion, all 24 MA lines investigated had a significant lower or higher selection coefficient than the control lines in at least one condition, in accordance with the accumulation of spontaneous mutations in each MA line and a variation in the effects of spontaneous mutations in different environments.

## Discussion

### No fitness decrease in three out of four species: no mutations or mutations with no fitness effects?

Except for *O. tauri*, most MA lines did not show any evidence of fitness decrease during the experiment. This is despite running the experiment with a low average effective population size of around eight individuals, at maximum, over 265–272 generations. Several factors might explain the absence of fitness decrease in most MA lines.

First, it could be due to a very low mutation rate. The low mutation rate could be a result of large effective population sizes in these species, that enable selection for lower mutation rate, limiting the appearance of deleterious mutations (Lynch 2010; Sung *et al.* 2012). Nevertheless, it is possible to estimate a minimum mutation rate, assuming that a significant fitness difference between the controls and the MA lines might be the result of at least one mutation. Since each of the MA lines has a significant fitness difference with the control in at least one condition, this corresponds to nine mutations for *O. mediterraneus*, seven for *M. pusilla*, and eight for *B. prasinos*. Depending on the number of generations and the genome size, the minimum mutation rate is thus 2.72^−10^ mutations/site/generation for *O. mediterraneus* (*i.e.*, 0.0037 mutations/genome/generation), 1.75^−10^ for *M. pusilla* (*i.e.*, 0.0037 mutations/genome/generation), and 2.52^−10^ for *B. prasinos* (*i.e.*, 0.0038 mutations/genome/generation). These estimates are consistent with estimates in other unicellular organisms, like *C. reinhardtii* (Ness *et al.* 2012) with 2.08^−10^ mutations/site/generation, or *S. cerevisiae* with 3.30^−10^ mutations/site/generation (Lynch *et al.* 2008), *Schizosaccharomyces pombe* with 2.00^−10^ mutations/site/generation (Farlow *et al.* 2015), *Burkholderia cenocepacia* with 1.33^−10^ mutations/site/generation (Dillon *et al.* 2015), or *E. coli* with 2.45^−10^ mutations/site/generation (Lee *et al.* 2012). Thus, fitness assays suggest that the minimum mutation rates of our strains are not lower than those in other species and are close to the constant mutation rate proposed by Drake (Drake 1991), that is *U* = 0.0033 in microorganisms.

Second, our measure of fitness may not be well suited to detect the effect of mutations. In a MA experiment in *D. discoideum*, Hall and coworkers followed eight fitness traits, and showed that two of them did not decrease (Hall *et al.* 2013). We measured fitness as the rate at which the population increased over the 2 wk period between two bottlenecks. Most of the species tend to divide once a day, in rhythm with the natural LD cycle, and so this is probably a robust character, particularly under the benign lab conditions. Likewise, cell death may not occur very often under laboratory conditions. However, the fact that all MA lines show significant fitness differences with the control lines under stressful conditions suggests that at least some mutations with fitness effects have occurred. Indeed, the fitness effects of mutations change across environments. Previous mutation experiments in *Caenorhabditis* (Baer *et al.* 2006) and *D. melanogaster* (Fry *et al.* 1996; Fry and Heinsohn 2002) suggest that mutational parameters change, as expected because of GxE interactions.

Third, although all of these species are usually haploid, some lines may have become diploid during the experiment, which may have masked the effects of some deleterious mutations. However, we would expect an increase of cell size with ploidy change, but this was not observed by flow cytometry.

Finally, the duration of the experiment may not have been sufficient to detect the effects of deleterious mutations. A decrease of fitness was observed in *O. tauri*, which was allowed to accumulate mutations over a longer period than the other three species (512 generations as compared to the 272 and 265 in the other species). Indeed, recent MA experimental studies in *C. reinhardtii* (Morgan *et al.* 2014) and *D. discoideum* (Hall *et al.* 2013) reported a decrease in fitness with similar effective population sizes and higher numbers of sequential generations (N_e_ = 6.5 during ∼1000 generations, and N_e_ = 7.5 during ∼994 generations, respectively). However, increasing the number of sequential generations beyond 200 was not possible: in *B. prasinos* and *M. pusilla*, the MA experiments had to be stopped as a consequence of the high line loss at each bottleneck. The number of lines lost was leading to a stagnation of the total number of independent generations in the experiments. Line loss occurred at each bottleneck from the start of the experiment and there was no trend (increase or decrease) in the number of lines lost with time. There are three possible explanations for line loss.

First, it could be due to sampling error, since single cell transfer cannot be checked by eye or light microscopy due to small cell size. The probability of sampling one single cell from a volume follows a Poisson distribution and the probability of sampling no cell is thus 0.37. To overcome this high rate of loss, our experimental procedure was to sample a volume of culture predicted by flow cytometry to contain 10 cells and divide this into six wells of a culture plate (see *Materials and Methods*). The probability of line loss is thus smaller than 10^−2^ in all experiments (Table 2). Coefficients of variation between 0.4–0.5 are needed to account for the observed line loss. However, since cytometry counts and pipetting errors are below 1%, it is highly unlikely that the sampling procedure is responsible for the observed level of line loss.

Second, line loss may be the consequence of lethal mutations or strong selection imposed by the experiment. If the experiment was associated with selection, we would expect the growth rates from the control cultures, reinoculated at the same time with 100 cells, to increase over the course of the experiment. There is no evidence for this in any experiment. On the other hand, if lethal mutations are responsible for the line loss, the rate of lethal mutations per generation can be estimated by the proportion of lost lines divided by the number of generations and is 0.025 and 0.019 per genome per generation in *B. prasinos* and *M. pusilla*, respectively. Compared to the known spontaneous mutation rates in other microorganisms (Drake 1991) and the estimations above, these lethal mutation rates would be five to sevenfold higher than the spontaneous mutation rates reported above. This corresponds to lethal mutation rates that are too high to be viably supported by a population.

A third hypothesis is that line loss is not the consequence of cell death but the consequence of the absence of cell division. In lab conditions, living cells usually engage in cell division at the end of the day, after light exposure, provided nutrients are available. Without bottleneck to one single cell, line loss in culture maintenance is exceptional. However, if cell division is triggered by an environmental factor produced by the culture, it may be halted as a consequence of the reinoculation step of one single cell. Consistent with this hypothesis, we observed that lost lines were transferred from significantly smaller volumes; from 2 µl on average, while maintained lines have been transferred from 4 µl, on average, for *M. pusilla* and *B. prasinos* (Student’s test, p-values < 0.001 and < 0.01, respectively). The difference in line loss rates between species could thus be the consequence of a difference in dependence of cell division to an environmental factor, lost during the reinoculation step. This environmental factor may be a metabolite produced by the culture, *e.g.*, a phytohormone (Bartel 1997; Piotrowska-Niczyporuk and Bajguz 2014). This high level of line loss reveals a knowledge gap on the induction of cell division in nonmodel microorganisms and reduces the amount of data available for fitness estimates. However, it does not alter the growth rate estimates of the maintained lines or the estimations of mutations per generation.

### Increase or decrease of fitness under stressful conditions

Changes in environmental conditions clearly enable the detection of substantial variation in fitness between MA lines. This is as expected if the fitness effect of mutation changed between environments. The variance between the MA lines is greater than the variance between the control lines, suggesting that some mutations, not detected in MA standard conditions, have been fixed in our MA lines. The significant variation in fitness of some MA lines may be the result of several nonmutually exclusive factors.

First, stressful conditions might exacerbate already existing fitness differences (Kondrashov and Houle 1994), so the MA lines may have accumulated more slightly deleterious mutations than the control lines because they have smaller *N_e_*, but the overall difference in fitness between the MA and control lines is not detectable under the standard MA conditions. However, such differences in fitness might be detectable in a stressful environment because the selection intensity changes (Martin and Lenormand 2006). A change in selection intensity might come about through a change in the environment (Fry and Heinsohn 2002; Rutter *et al.* 2012), or a change in the effect of an allele, for example by a change in gene expression. In another green algae, *C. reinhardtii*, Kraemer and coworkers also highlight the effects of stress on the amplification of deleterious mutations and their impact on fitness (Kraemer *et al.* 2015).

Second, the fixation of mutations, particularly slightly deleterious mutations, is faster in the MA lines because they have smaller *N_e_*. As a consequence, these slightly deleterious mutations, which could become advantageous in a novel environment, can accumulate in the MA lines but not in the controls. They may thereby increase the fitness in some of these MA lines. In addition, both the control and MA lines have accumulated mutations that are neutral under the original conditions but deleterious under the stressful conditions, causing the fall of fitness among MA lines.

### Conclusion

We investigated the accumulation of mutations in four marine green picoalgae. Despite a modest number of sequential generations per MA line, we found evidence for a variation in fitness effects of spontaneous mutations from benign to stressful environments. This allowed us to estimate a minimum per genome mutation rate of 0.0037.

## Supplementary Material

Supplemental Material
