# Supplementary material for: Fitness Effects of Spontaneous Mutations in Picoeukaryotic Marine Green Algae
Source: G3 (Bethesda). 2016 May 10;6(7):2063–71. doi: 10.1534/g3.116.029769 (PMC4938659; doi:10.1534/g3.116.029769)
Supplement: Supplemental Material [file supp_6_7_2063__index.html]

Fitness Effects of Spontaneous Mutations in Picoeukaryotic Marine Green Algae — Supplemental Material 

# Fitness Effects of Spontaneous Mutations in Picoeukaryotic Marine Green Algae

## Supplemental Material for Krasovec *et al.*, 2016

**Files in this Data Supplement:**

- Table S1 - Normalized *Gr* from *Micromonas pusilla* MA lines that survived since the beginning to the end of the mutation accumulation experiment, at each bottleneck, from 14 to 302 days. (.xlsx, 10 KB)
- Table S2 - Normalized *Gr* from *Ostreococcus mediterraneus* MA lines that survived since the beginning to the end of the mutation accumulation experiment, from 14 to 294 days. (.xlsx, 12 KB)
- Table S3 - Normalized *Gr* from *Bathycoccus prasinos* MA lines that survived since the beginning to the end of the mutation accumulation experiment, at each bottleneck, from 14 to 224 days. (.xlsx, 10 KB)
- Table S4 - Normalized *Gr* from *Ostreococcus tauri* MA lines that survived since the beginning to the end of the mutation accumulation experiment, at each bottleneck, from 140 to 378 days. (.xlsx, 12 KB)
- Table S5 - Average of *G* of MA lines and control of *Micromonas pusilla* for each environmental test. (.xlsx, 10 KB)
- Table S6 - Average of *G* of MA lines and control of *Bathycoccus prasinos* for each environmental test. (.xlsx, 10 KB)
- Table S7 - Average of *G* of MA lines and control of *Ostreococcus mediterraneus* for each environmental test. (.xlsx, 10 KB)
- Table S8 - *G* of the 24 controls of *B. prasinos* during the MA experiment. (.xlsx, 13 KB)
- Table S9 - *G* of the 24 controls of *M. pusilla* during the MA experiment. (.xlsx, 13 KB)
- Table S10 - *G* of the 24 controls of *O. tauri* during the MA experiment. (.xlsx, 12 KB)
- Table S11 - *G* of the 24 controls of *O. mediterraneus* during the MA experiment. (.xlsx, 13 KB)
